# The Danish-American Research Exchange (DARE): a cross-sectional study of a binational research education program

**DOI:** 10.1186/s12909-023-04002-z

**Published:** 2023-02-06

**Authors:** Kala M. Mehta, Karin Lottrup Petersen, Steve Goodman, Henrik Toft Sørensen, Martin Bøgsted, Jeppe Dørup Olesen, Sylvia Burks, Richard E. Shaw, Jens Dahlgaard Hove, Jakob Ousager, Carlos Milla, Vibeke Andersen, Niels Ejskjær, Vibeke Brix-Christensen, Shomit Ghose, Andreas Kjær, Peter V. Chin-Hong

**Affiliations:** 1 Department of Epidemiology and Biostatistics, University of California, San Francisco, CA USA; 2 Independent Consultant, Copenhagen, Denmark; 3 Health Research and Policy, Stanford, CA USA; 4 Department of Clinical Epidemiology, Aarhus University and Aarhus University Hospital, Aarhus, Denmark; 5 Department of Clinical Medicine, Aalborg University, Aalborg, Denmark; 6 Aarhus Institute of Advanced Studies, Aarhus, Denmark; 7 Burks Johansson LLP, Palo Alto, CA USA; 8 California Pacific Medical Center, San Francisco, CA USA; 9 Department of Cardiology, Copenhagen University Hospital Hvidovre, Copenhagen, Denmark; 10 Faculty of Health Sciences, University of Southern Denmark, Odense, Denmark; 11 Department of Pediatrics-Pulmonary Medicine, Stanford, Stanford, Calfornia USA; 12 Research Unit for Molecular Diagnostic and Clinical Research, IRS-Center Soenderjylland, Institute of Molecular Medicine, University of Southern Denmark, Odense, Denmark; 13 Steno Diabetes Center North Denmark and Department of Endocrinology, Aalborg University Hospital, Aalborg, Denmark; 14 Department of Pediatrics, Copenhagen University Medical School, Copenhagen, Denmark; 15 Department of Engineering, University of California Berkeley, Berkeley, CA USA; 16 Department of Clinical Physiology, Nuclear Medicine & PET, Rigshospitalet and University of Copenhagen, Copenhagen, Denmark; 17 Department of General Internal Medicine, University of California San Francisco, San Francisco, CA USA

**Keywords:** Binational, Medical student, Denmark, United States, Training programs

## Abstract

**Background:**

Most medical educational programs emphasize clinical observation or clinical skill acquisition, fewer focus upon research. The Danish-American Research Exchange (DARE) program, sponsored by the Lundbeck Foundation, is unique in that the medical student initiates biomedical research collaboration between Danish and US medical institutions. To achieve this, Danish medical students (DARE students) conduct binational mentored research projects while based in the United States for 10 months. In addition, DARE students are introduced to interdisciplinary thinking about how to develop ultra-low-cost healthcare interventions through the ‘$10 Challenge’.

**Methods:**

We conducted a cross-sectional study of DARE alumni over five consecutive years (2015–2020, *n* = 24). Research metrics included completion of a research project, primary authorship, and co-authorship of publications. The number of publications, prior to and after the DARE program were enumerated. For the first four cohorts, graduation from medical school and acceptance or intention to enter a joint MD-PhD program also were assessed. Two focus groups were conducted using constructivist grounded theory. Discussions were transcribed, redacted, and coded using Dedoose software.

**Results:**

DARE Medical students were 31.2 years (range 24–35), the majority were women (67%;16/24). The majority (17/24;71%) completed a first author publication in a peer-reviewed journal with a median of 3.9 per DARE alumnus. DARE alumnus reported increased proficiency in biostatistics, epidemiology, coding and public speaking as well as stronger research qualities in creativity, critical thinking, comfort in approaching scientist in both the US and Denmark (*p* < 0.001 for all). Qualitative key themes included: increased confidence, a deepening of research inquiry and linkage to a research network.

**Conclusions:**

Preliminarily, this study suggests that medical students can initiate binational collaboration in medicine. Benefits include research productivity, intention to pursue academic medical careers, as well as positive impacts on motivation. This medical student-initiated research model lays the groundwork for using this model across other country pairs to promote binational collaboration.

**Supplementary Information:**

The online version contains supplementary material available at 10.1186/s12909-023-04002-z.

## Background

Cross-pollination of ideas and research collaboration among medical doctors in different countries have advanced scientific inquiry since antiquity. The practice of multinational collaboration ranges from spontaneous informal meetings at medical conferences to more formal ‘sister’ collaborations between hospitals and medical systems across countries [1].

Specific pairings at the institutional level arise to promote clinical exposure, knowledge transfer, development of surgical or other skills, and dissemination of best practices in medicine. Historically, a primary reason for binational programs has been to strengthen training, e.g., by teaching specific skills to practicing MDs in low-resource settings. The Doris Duke Charitable Foundation and the Fogarty International Center fund such programs between medical institutions of the United States and medical doctors in Africa and Asia [2–6].

The DARE program, sponsored by the Lundbeck Foundation, is a new and novel binational collaboration between two high-income countries (US and Denmark), it focuses on clinical research with an added component via exposure to an entrepreneurial environment. The current paper presents the characteristics of the DARE program after five years, as well as research indicators and changes in thinking among DARE program participants, all of whom are Danish medical students.

## Methods

### Aim and design

This is a cross sectional study which aims to describe the characteristics and outcomes of a novel binational program (DARE) over 5 years (2015–2020).

### Setting and characteristics of participants

The DARE program draws from 4 university-affiliated medical schools in Denmark, bringing students to University of California San Francisco (UCSF) and Stanford University School of Medicine. The Danish schools are Copenhagen University, University of Southern Denmark, Aarhus University, and Aalborg University. The program is funded by the Lundbeck Foundation. The Innovation Centre Denmark in Silicon Valley (ICDK), a collaboration between the Danish Foreign Ministry and the Ministry of Higher Education and Science, is the fiscal agent.

### Selection criteria

DARE applicants submit an application proposing a binational mentorship team, with one mentor from Denmark and another from the US. Faculty must be full time. The mentors must work in similar or compatible fields, but do not need previous history of collaboration. The mentorship team and the student develop a novel research project that extends the professors’ primary research by incorporating data, patients, techniques or materials only available from one side or the other, or by replicating a study in a different setting.

### Application process

Applicants first submit a letter of intent (LOI) comprised of a short research proposal, description of their mentorship team and their curriculum vitae, and certification that students meet the Danish medical schools’ requirements to take a year away from primary medical study to pursue research. Eligible students are asked to submit a full application detailing the research project and containing the curriculum vitae of both the US and Danish mentors, as well as a short video describing themselves and their projects. Interviews were conducted by several members of a 12-person selection committee. Selection criteria include the strength of the mentorship team, the scientific merit of the project, the feasibility of completing the project in 10 months, and the candidate’s commitment to research and research potential.

### Participants

Beginning in 2015, the DARE program (initially the Lundbeck Foundation Clinical Research Fellowship) has sponsored five medical students per year. Here we report on outcomes from the first 24 students, in 5 cohorts from 2015–2020. The program is executed for 10 months per year, from August 1 – June 1 each cycle.

### Program description

The DARE scholars participate in a 20-h graduate medical education course entitled ‘Designing Clinical Research’ taught at UCSF over the summer. For the subsequent 9 months, the scholars work on a mentored clinical research project for 40 h per week in a research team or laboratory. During the 9 months, they also take part in an ongoing biweekly 2-h sessions run by DARE faculty, comprising a journal club, presentation of works in progress, topics in study design and analysis, and career development skills. The Institutional Review Board at the University of California, San Francisco approved this study.

### Fostering innovative thinking: the “$10 Challenge”

Another unique aspect is that DARE scholars gain exposure to innovative thinking by engaging in the “$10 Challenge.” This is an activity outside of their main clinical research project. The goal of the challenge is to develop a way to address a large global public health problem that costs no more than $10 per patient per use. The DARE students lead teams of US medical students and learners from Business/Entrepreneurship, Bioengineering/ Technology, and Marketing/Innovation to create a total cohort of 25, split into 5 separate teams. Each team works on their project in six monthly meetings, guided by a seasoned professional entrepreneur. Participants learn from venture capitalists, entrepreneurs, and Silicon Valley professionals how to identify a target market, evaluate manufacturing resources, assess market size, devise market strategy (including social media marketing), identify sales and distribution channels, and legal matters including protecting intellectual property.

### Measures

All students in the DARE program were asked to complete an exit survey, a supplemental survey and two in-person focus group discussions in Summer, 2020. Program measures included publication of a paper in a peer-reviewed scientific journal, a program requirement. Authorship was categorized into primary or co-authorship, and publications were categorized separately if they had evidence of binational collaboration. We also counted students’ oral and poster meeting presentations as a primary author. The number of academic awards per student was also calculated prior to, during, and after the DARE program. The first three cohorts of students were asked whether they had graduated from medical school, assumed a primary position as a practicing physician, and about their acceptance/intent to pursue a joint MD-PhD degree.

We used a quantitative and a qualitative approach to understand how the DARE program impacted the students’ attitudes and skills related to research and innovation. We asked DARE alumni to rate their proficiency in research skills such as public speaking, English speaking, epidemiology, biostatistics and coding, efficiency, creativity, critical thinking, working long hours, comfort with a large workload, and comfort approaching scientists from the US and Denmark prior to DARE and after DARE on a Likert 5-point scale.

Through focus group discussions to address these same domains, data collection and analyses were informed by principles of constructivist grounded theory (CGT) [7]. These focus groups lasted 1.5 h and the discussions structured by an interview guide. Due to the COVID-19 pandemic, we conducted individual structured key informant interviews utilizing the same guide for the focus groups in the prior two years. Discussions were recorded and transcribed, redacted, and coded using Dedoose software (Dedoose.com, Los Angeles, US). Applying principles of CGT, the focus group transcript was inductively analysed using open coding by two members of the research team. Preliminary codes were cross-checked and discrepancies discussed until consensus was reached. The codes were organized into themes and relationships.

### Statistical analyses

We summarized descriptive characteristics of the student scholars including age, sex, race/ethnicity, grade average prior to entering DARE and their Danish Medical Institution. We also described project characteristics, that is, the type of project (clinical research or translational/basic science) and discipline within medicine. We described research metrics for all 24 scholars, overall, and stratified by age (above and below 31.2), gender, type of project, whether the scholar had prior publications (none compared to more than one). We used paired t-tests to examine differences prior to DARE compared to after DARE.

## Results

The average age of the 24 medical students at entry was 31.2 years (range 24–35), 67% were women (16/24), and 100% were White/Caucasian. 54% went to Copenhagen Medical School (13/24), followed by Aarhus (4/24, 17%), Aalborg (4/24, 17%) and Southern University Denmark (3/24, 13%). 54% were at UCSF (13/24), (38%) at Stanford (9/24) and 8% (2/24) students had mentors at both institutions. All students responded (Table 1).

**Table 1:** Characteristics of 24 Danish American Research Exchange (DARE) students, 2015–2020

Factor	Level	Value
Mean Age (years, standard deviation)		31.2 (2.3)
Sex	Female	16 (67%)
	Male	8 (33%)
Race	White/Caucasian	24 (100%)
Danish Institution (proportion, %)	Aalborg University	4 (17%)
	Aarhus University	4 (17%)
	Copenhagen University	13 (54%)
	Sydansk University	3 (13%)
Mean grade average in units (0–12 scale, 12 = high), during Bachelors (standard deviation)		8.9 (1.8)
Mean grade average in units (0–12 scale, 12 = high), during Masters (standard deviation)		10.0 (1.1)
US Institution (proportion, %)	UCSF	13 (54%)
	Stanford	9 (38%)
	UCSF/Stanford	2 (8%)
Cohort	2015–2016	5 (21%)
	2016–2017	5 (21%)
	2017–2018	5 (21%)
	2018–2019	5 (21%)
	2019–2020	4 (17%)
Completed Manuscript requirement	Yes	17 (71%)
First Author publications, mean (SD)		2.1 (2.9)
Collaborative Author publications, mean (SD)		1.8 (2.4)
Total Publications, mean (SD)		3.9 (4.7)
Publications Attributable to DARE, mean (SD)		1.3 (1.7)
Publications prior to program, mean (SD)		1.5 (2.7)
Publications post program, mean (SD)		3.1 (3.8)
Binational publications, mean (SD)		.71 (.75)
Type	Epidemiology/Clinical Data	13 (54%)
	Translational Science	11 (46%)
Discipline	Anesthesiology	1 (4%)
	Cardiology	4 (17%)
	Endocrinology	2 (8%)
	Gastroenterology	2 (8%)
	Head and Neck	1 (4%)
	Hepatology	1 (4%)
	Nephrology	2 (8%)
	Neurology	1 (4%)
	Nuclear Medicine	2 (8%)
	Oncology	3 (13%)
	Orthopedic Surgery	1 (4%)
	Pediatrics	1 (4%)
	Psychiatry	1 (4%)
	Rheumatology	2 (8%)

Most students did clinical research (54%, 13/24), followed by translational/basic science projects in a ‘wet’ lab (46%, 11/24). Research projects covered fourteen medical areas (Supplemental Fig. 1a, b).

Seventy one percent of students completed the first-author requirement; 58% percent of students had a paper indicating a binational collaboration, that is, a publication with both mentors. (Table 1 and Appendix 1) Scholars averaged 3.9 publications per DARE alumnus (median 2, IQR 0.5–7). Number of publications increased from a mean of 1.5 (2.7) pre-Dare to a mean of 3.1 (3.8) post DARE, most attributable to the program. There were no statistically discernible differences by age, sex, type of project or prior publications.

Among students in the first three DARE cohorts, one student has earned a PhD (4%), four alumni are in a PhD program (17%), combined (5/24 21%). Of the remaining students, 13 (54.2%) indicated that they intend to pursue a PhD program in the future (Supplemental Fig. 2).

DARE scholars reported increased proficiency in several domains after the DARE program, all with *P* < 0.01, measured on 5-point scales: public speaking (+ 1.5 points), English speaking (+ 0.67 points), Epidemiology (+ 1.42 points); Biostatistics (+ 1.25); Coding (+ 1.54 points). They also reported increased creativity (+ 0.375, *p* = 0.009); critical thinking (+ 0.79); comfort with approaching Danish scientists (+ 1.5); and comfort with approaching US scientists (+ 1.9). Evidence for improved efficiency was modest (+ 0.29, *p* = 0.07); ability to work long hours (+ 0.21, *p* = 0.17); or comfort with a large workload (+ 0.25, *p* = 0.17) (Table 2).

**Table 2:** Self-rating of Proficiency in Research Skills and Research Work Ethic for DARE scholars (*n* = 24)

	Prior to DARE	After DARE	*P*-value
Public Speaking	1.83 (.76)	3.33 (.64)	*P* < 0.0001
English Speaking	3.13 (.68)	3.79 (.41)	*P* < 0.0001
Epidemiology	2.04 (.75)	3.46 (.59)	*P* < 0.0001
Biostatistics	1.79 (.66)	3.04 (.69)	*P* < 0.0001
Coding	1.08 (1.14)	2.63 (1.28)	*P* < 0.0001
Self rating of work ethic, mean (SD) rating 1–5 (1 = least strong 5 = most strong)			
I am more efficient	2.96 (.69)	3.25 (.75)	*P* = 0.0695
I am creative	2.79 (.83)	3.16 (.76)	*P* = 0.0093
I have critical thinking skills	2.95 (.55)	3.75 (.44)	*P* < 0.0001
I work long hours	2.96 (.69)	3.17 (.76)	*P* = 0.1703
I am comfortable with a large workload	3.13 (.79)	3.17 (.76)	*P* = 0.167
I am comfortable approaching scientists from Denmark	2.38 (.97)	3.92 (.28)	*P* < 0.0001
I am comfortable approaching scientists from the US	1.88 (.90)	3.75 (.44)	*P* < 0.0001

Self-rating of proficiency, mean (SD) rating 1–5 (1 = least proficient 5 = most proficient)

*P*-values calculated as paired t-tests

The qualitative portion of this study sought to understand how the DARE program impacted the students’ thinking (Supplemental Table 1). They distinguished between their experience in the main (clinical research) program and the $10 Challenge experience. Key themes related to the clinical research component are illustrated by quotations from the focus groups, such as increased confidence:

“It really changed the way I view the world and my approach to research, which became much more creative and structured. I gained confidence in my own ideas, my way of doing things.” [about the DARE program]

Another key theme that emerged was being forced to delve into a topic more deeply than previously:

“in general at [US University] that they really kept digging, kept digging, kept digging, where we would stop earlier on the Danish side.” [about the DARE program]

DARE fellows felt that they built a community of like-minded peers, which continued as DARE alumni:

“really missing have some peers to actually discuss problems with. And I used you guys a lot and also the American students, I got inspired from those meetings”

Broader results from the qualitative analyses are shown in Supplemental Table 1.

## Discussion

The DARE program represents a unique method for promoting binational partnership between trainees and senior faculty from two countries. After completing five years of the program, 24 DARE alumni had 97 peer-reviewed articles cited in PubMed, 20% indicating binational collaboration. DARE medical students also were active in presenting their work at international conferences, averaging about two conferences per student. Scholars reported increased proficiency in epidemiology and biostatistics, public speaking, biostatistics coding and English proficiency as well as gains in creativity, critical thinking, and comfort in approaching other scientists. Qualitative emergent themes were increased confidence, digging ‘deeper’ into their science, and building a community of like-minded peers. Students reported that their DARE experience changed their thinking well after the program.

We compared our results to a study of Danish medical students who completed a research year in Denmark between 2004 and 2013, where approximately 36% published a first-author paper [8]. In comparison, 71% of DARE medical students published a first-author paper, almost double. Also, DARE students published approximately 2.5 publications per year, compared to 1.1 for MD-PhD students in Denmark. This suggests that DARE alumni outperform students roughly 4 years further along in their education [9].

The research productivity and intention to pursue research was similar between DARE and the Fogarty International Fellows program [6, 10] and compared to the Doris Duke Charitable Foundation Medical Student program, though data from the latter program are now approximately 15 years old [2, 5]. The difference between these programs is that the DARE scholar initiated the research project and binational collaboration in all instances.

Entrepreneurial concepts such as “design thinking” are relatively new in medical education, [11, 12] and were particularly new for these students. This led students to affirm the themes of increased scientific risk-taking, enhanced persistence, increased problem-solving skills, and heightened confidence.

Some limitations deserve comment. The program is small, and inferences from just 24 students in the first 5 cohorts should be interpreted with caution. In addition, we have had a relatively short follow up, from 1 to 4 years. It was also difficult to construct an ideal control group. Averaged data for Danish medical students, may be available through the Danish Ministry of Science and Education in the future, albeit complicated by varying durations of medical school training. Better controls would be research year participants who stay in Denmark, but publications and other outcomes are either not systematically tracked at the four Danish medical schools or are not publicly available.

## Conclusions

In summary, the DARE binational medical program described here is a unique model of how students can initiate binational collaboration. It builds on the resources of four Danish medical schools and two medical campuses in the United States. Binational collaborations involving 24 medical students have been documented thus far, including a robust record of publications and international presentations as well as increased confidence, digging ‘deeper’ into their science, and building a community of like-minded peers. Future work in this field is needed to better define how to measure binational collaboration impacts the mentors and how it benefits the academic medical institution as a whole [13].

## Supplementary Information

**Additional file 1: Figure 1.** Type (1a) and Discipline (1b) of Post Program Publications; Danish American Research Exchange (DARE) students, 2015-2020.

**Additional file 2: Figure 2.** Academic Degrees of Danish American Research Exchange (DARE) Students, 2015-2020.

**Additional file 3**: **Supplemental Table 1.** Qualitative data from DARE medical student alumni collected at two focus group meetings. **Appendix 1.** References for publications of DARE Fellows and Alumni, 2015-2020, Cohorts 1-5.

## Data Availability

The datasets generated and/or analyzed during the current study are not publicly available because they are part of an educational program and potentially contain identifiable information but are available from the corresponding author in a de-identified form on reasonable request.

## Abbreviations

DARE: Danish American Research Exchange
US: United States
*e.g*.: Exempli grata, Latin such as
MD-PhD: Medical Doctor-Doctor of Philosophy
PhD: Doctor of Philosophy
MD: Medical Doctor
*i.e*.: *id est, Latin* That is
CGT: Constructivist grounded theory
SD: Standard Deviation
SE: Standard Error of the Mean
IQR: Interquartile Range
