# Supplementary material for: The Danish-American Research Exchange (DARE): a cross-sectional study of a binational research education program
Source: BMC Med Educ. 2023 Feb 6;23:96. doi: 10.1186/s12909-023-04002-z (PMC9902060; doi:10.1186/s12909-023-04002-z)
Supplement: Supplementary file 1 — Additional file 1: Figure 1. Type (1a) and Discipline (1b) of Post Program Publications; Danish American Research Exchange (DARE) students, 2015-2020. [file 12909_2023_4002_MOESM1_ESM.pdf]

Figure 1: Type (1a) and Discipline (1b) of Post Program Publications; Danish American Research Exchange (DARE) students, 2015-2020.

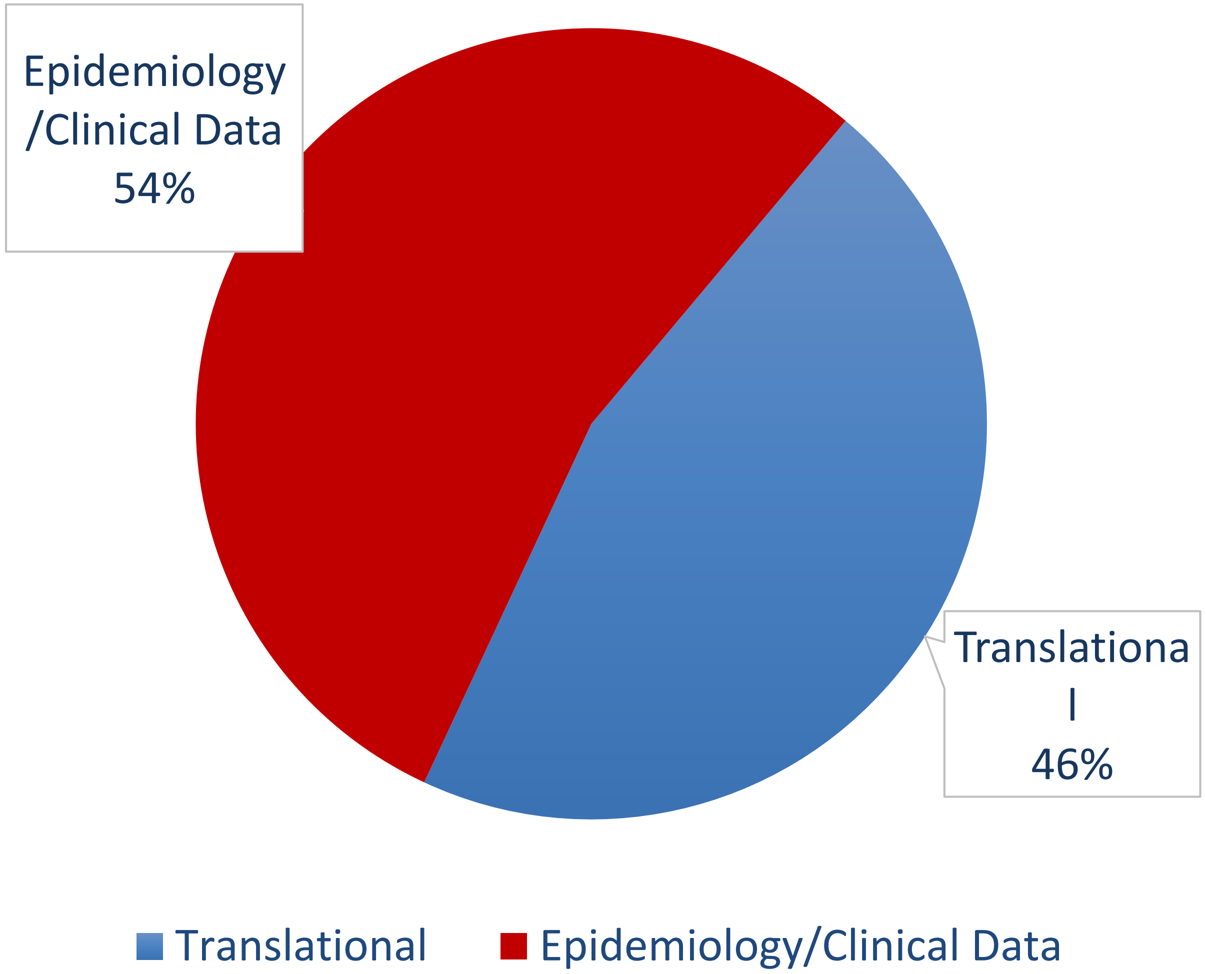

Figure 1a Type of Publication

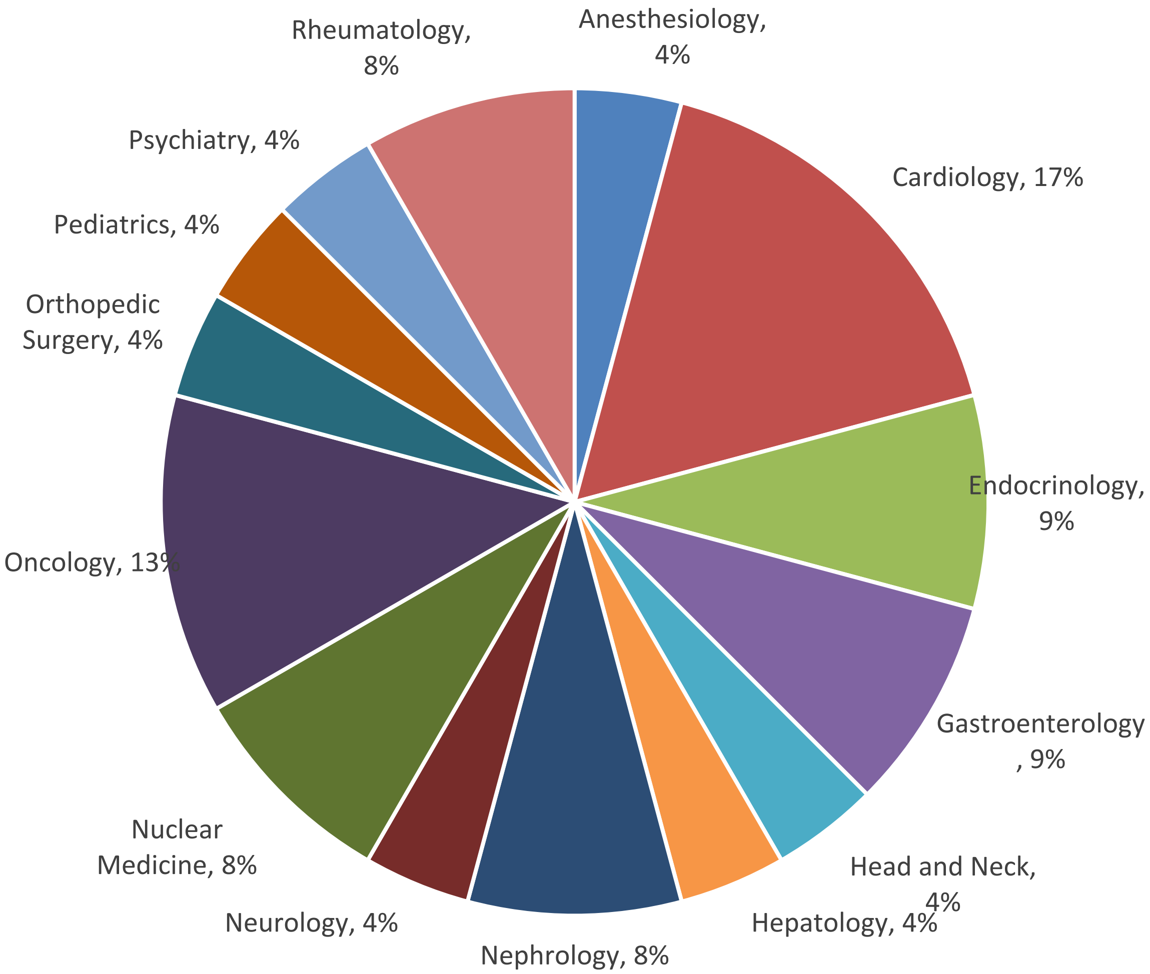

Figure 1b Discipline of Publication
