# Supplementary figures and images for: The Danish-American Research Exchange (DARE): a cross-sectional study of a binational research education program
Source: BMC Med Educ. 2023 Feb 6;23:96. doi: 10.1186/s12909-023-04002-z (PMC9902060; doi:10.1186/s12909-023-04002-z)

Figure 2: Academic Degrees of Danish American Research Exchange (DARE) Students, 2015-2020.

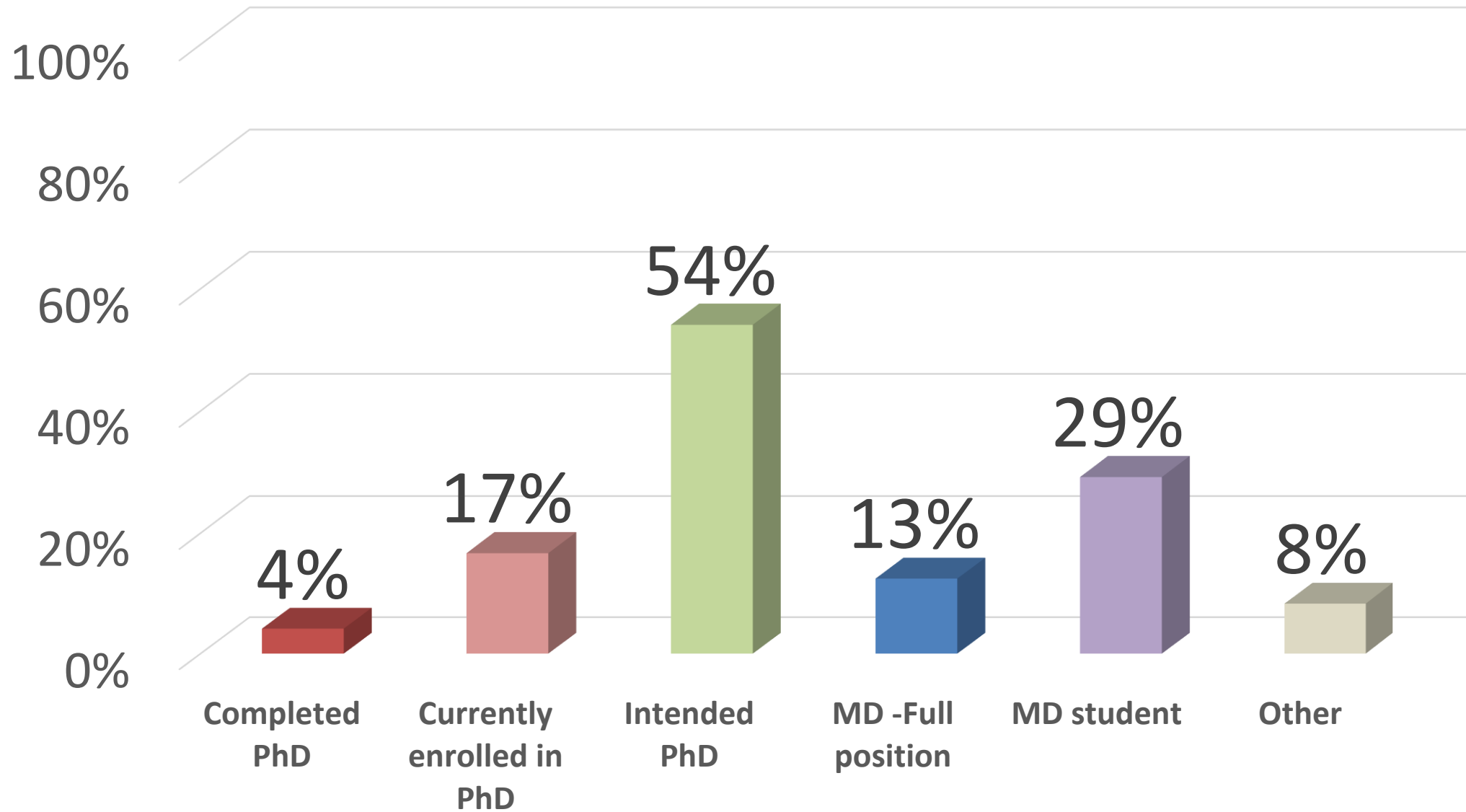

Supplement: Supplementary file 2 — Additional file 2: Figure 2. Academic Degrees of Danish American Research Exchange (DARE) Students, 2015-2020. [file 12909_2023_4002_MOESM2_ESM.pdf]
