# Supplementary material for: The Danish-American Research Exchange (DARE): a cross-sectional study of a binational research education program
Source: BMC Med Educ. 2023 Feb 6;23:96. doi: 10.1186/s12909-023-04002-z (PMC9902060; doi:10.1186/s12909-023-04002-z)
Supplement: Supplementary file 3 — Additional file 3: Supplemental Table 1. Qualitative data from DARE medical student alumni collected at two focus group meetings. Appendix 1. References for publications of DARE Fellows and Alumni, 2015-2020, Cohorts 1-5. [file 12909_2023_4002_MOESM3_ESM.zip › Supplementary Table 1_ESM.docx]

**Supplemental Table 1:** Qualitative data from DARE medical student alumni collected at two focus group meetings.

| Key Theme: Changed overall approach increased confidence | |
| --- | --- |
| DARE fellowship | $10 Challenge |
| I really learned a lot in San Francisco. It really changed the way I view the world and my approach to research, which became much more creative and structured. I gained confidence in my own ideas, my way of doing things.  Really inspiring, and it taught me that with hard work and dedication and the right kind of support, you can really achieve almost anything you set your mind to.  You become an ambassador for your field  That mistakes are not failure and that's very different from Denmark where I often find a barrier towards doing anything at all, it's that, "Oh no, it might go wrong." So we take the other path, we always have to stay clear and stay safe and never take any chances. | I suddenly have some new knowledge I didn't have before at all. So for example, I met with a lawyer and a consultant and their friends are doing this totally different stuff, but I feel like, even though a lawyer is both far away from medicine and business, I feel like I can speak to these people in a different way because I learned something [in $10 challenge]. And I can understand their world in a different way and I think it gives me a broader network in the end |
|  | I think the biggest and most important impact that the DARE program has had on me is the whole mindset of what is actually possible. |
| Key Theme: Going ‘deeper’ into science than before | |
| the fellowship gave you time to really go in depth.  got that feeling from the research environment. And that close collaboration having a full-year to really engage with this, that really made the difference  I think I learned a lot from the discussions we had from everyone in the lab trying to make each other better  forces to think about why did we do this | somehow indirectly applied it in both my medical career and in research. The whole thought process and the whole finding what is the problem and thinking about a solution and who do I have to involve in order to get this solved. Being goal-orientated and solution-oriented, but really, I don't know, it changed a lot for me, actually. I became a better student and definitely a better researcher and doctor somehow. |
| I came home and the research group I was attached to in Denmark was like: ‘You almost did a Ph.D. in ten months’. So, I think it was way bigger. It was a lot. I think they were very ambitious. |  |
| in general at X that they really kept digging, kept digging, kept digging, where we would stop earlier on the Danish side.  just trusting process as XX said, I think at some point it will work out if you try enough times. You'll get something eventually. | when you're out there, you see all of those doctors [in DANISH] that complain, "This doesn't work, this doesn't work." And I'm more like, "Okay, what can we do to fix it?" I'm having a completely different mindset and really trying to take a deep breath |
| Key Theme: Developing a community of researchers | |
| I was really missing have some peers to actually discuss problems with. And I used you guys a lot and also the American students, I got inspired from those meetings. |  |
| The lab [in the US] was mostly on mice and basic lab and then the studies done in [Denmark] are mostly done with humans. It’s a bit of a different expertise area. If I had to draw on a similarity, they’re both working with muscles. There are differences between mice and humans. If I need expertise in one area, I’ll ask one lab and vice versa. It’s nice to have a broad field of experts that I could ask for different things. |  |

Supplemental table 1 legend: *Quotes redacted from direct transcripts of audiotaped conversations after transcription by Rev.com and coding using the Dedoose software system. Out of all alumni, 11/15 attended the first focus group in October 2018; 11/20 attended the second focus group in October 2019.

Focus group/Key informant interview Structured interview guide:

Think back to your fellowship year. What were some memorable experiences?

What were some memorable academic experiences? Can you give me some examples

Can you compare the project you did in the US to a typical project done at your university?

What do you think of the bi-national mentor teams? Did having a US and DK mentor help you in any way? If so, how?

When you came back to Denmark, what did you do? Did you notice any differences between your lab/mentor team in Denmark compared to the US? [try to get to changes in thinking, attitude, energy]

In terms of the fellowship year, how has it impacted you? What impact did DARE have in your career trajectory?

Have you participated in any leadership activities since returning to Denmark? What were they?

Can you speak specifically about the way the $10 challenge impacted you?

Are there any other ways you can describe the DARE experience in terms of your perspective or other thoughts you would like to share?

References

1. Aasbjerg K, Nørgaard CH, Vestergaard N, Søgaard P, Køber L, Weeke P, Gislason G, Torp-Pedersen C: **Risk of diabetes among related and unrelated family members**. *Diabetes research and clinical practice* 2020, **160**:107997.

2. Andersson C, Jorgensen ME, Martinsson A, Hansen PW, Gustav Smith J, Jensen PF, Gislason GH, Kober L, Torp-Pedersen C: **Noncardiac surgery in patients with aortic stenosis: a contemporary study on outcomes in a matched sample from the Danish health care system**. *Clinical cardiology* 2014, **37**(11):680-686.

3. Andersson C, Wissenberg M, Jorgensen ME, Hlatky MA, Merie C, Jensen PF, Gislason GH, Kober L, Torp-Pedersen C: **Age-specific performance of the revised cardiac risk index for predicting cardiovascular risk in elective noncardiac surgery**. *Circulation Cardiovascular quality and outcomes* 2015, **8**(1):103-108.

4. Andreasen C, Jorgensen ME, Gislason GH, Martinsson A, Sanders RD, Abdulla J, Jensen PF, Torp-Pedersen C, Kober L, Andersson C: **Association of Timing of Aortic Valve Replacement Surgery After Stroke With Risk of Recurrent Stroke and Mortality**. *JAMA cardiology* 2018, **3**(6):506-513.

5. Bachawal SV, Park JM, Valluru KS, Loft MD, Felt SA, Vilches-Moure JG, Saenz YF, Daniel B, Iagaru A, Sonn G *et al*: **Multimodality Hyperpolarized C-13 MRS/PET/Multiparametric MR Imaging for Detection and Image-Guided Biopsy of Prostate Cancer: First Experience in a Canine Prostate Cancer Model**. *Molecular imaging and biology* 2019, **21**(5):861-870.

6. Baekgaard JS, Eskesen TG, Moo Lee J, Ikast Ottosen C, Bennett Gyldenkaerne K, Garoussian J, Ejlersgaard Christensen R, Sillesen M, King DR, Velmahos GC *et al*: **Ketamine for rapid sequence intubation in adult trauma patients: A retrospective observational study**. *Acta anaesthesiologica Scandinavica* 2020, **64**(9):1234-1242.

7. Bernholm KF, Homoe AS, Meteran H, Jensen CB, Porsbjerg C, Backer V: **F eNO-based asthma management results in faster improvement of airway hyperresponsiveness**. *ERJ open research* 2018, **4**(4).

8. Bikle DD, Malmstroem S, Schwartz J: **Current Controversies: Are Free Vitamin Metabolite Levels a More Accurate Assessment of Vitamin D Status than Total Levels?** *Endocrinology and metabolism clinics of North America* 2017, **46**(4):901-918.

9. Bodker JS, Brondum RF, Schmitz A, Schonherz AA, Jespersen DS, Sonderkaer M, Vesteghem C, Due H, Norgaard CH, Perez-Andres M *et al*: **A multiple myeloma classification system that associates normal B-cell subset phenotypes with prognosis**. *Blood advances* 2018, **2**(18):2400-2411.

10. Brainin P, Olsen FJ, Lassen MCH, Bech J, Claggett B, Fritz-Hansen T, Folke F, Gislason GH, Biering-Sørensen T: **Postsystolic shortening on echocardiography as a gateway to cardiac computed tomography in patients with suspected stable angina pectoris**. *The international journal of cardiovascular imaging* 2020, **36**(2):309-316.

11. Christiansen MN, Andersson C, Gislason GH, Torp-Pedersen C, Sanders RD, Foge Jensen P, Jorgensen ME: **Risks of Cardiovascular Adverse Events and Death in Patients with Previous Stroke Undergoing Emergency Noncardiac, Nonintracranial Surgery: The Importance of Operative Timing**. *Anesthesiology* 2017, **127**(1):9-19.

12. Christoffersen C, Federspiel CK, Borup A, Christensen PM, Madsen AN, Heine M, Nielsen CH, Kjaer A, Holst B, Heeren J *et al*: **The Apolipoprotein M/S1P Axis Controls Triglyceride Metabolism and Brown Fat Activity**. *Cell reports* 2018, **22**(1):175-188.

13. Devereaux PJ, Duceppe E, Guyatt G, Tandon V, Rodseth R, Biccard BM, Xavier D, Szczeklik W, Meyhoff CS, Vincent J *et al*: **Dabigatran in patients with myocardial injury after non-cardiac surgery (MANAGE): an international, randomised, placebo-controlled trial**. *Lancet (London, England)* 2018, **391**(10137):2325-2334.

14. Federspiel C, Itenov TS, Thormar K, Bestle MH: **[Limited evidence for monitoring and treatment of hypophosphataemia in critically ill patients]**. *Ugeskrift for laeger* 2015, **177**(50):V07150610.

15. Federspiel CK, Itenov TS, Mehta K, Hsu RK, Bestle MH, Liu KD: **Duration of acute kidney injury in critically ill patients**. *Annals of intensive care* 2018, **8**(1):30.

16. Federspiel CK, Itenov TS, Thormar K, Liu KD, Bestle MH: **Hypophosphatemia and duration of respiratory failure and mortality in critically ill patients**. *Acta anaesthesiologica Scandinavica* 2018.

17. Federspiel CK, Liu KD: **Does Changing the Volume Matter? The Relationship of Urine Volume and Dialysis Intensity**. *Clinical journal of the American Society of Nephrology : CJASN* 2016, **11**(8):1321-1323.

18. Federspiel CK, Kelsen J, Fugleholm K: **Delayed Iatrogenic Intracranial Hypotension After Thoracotomy**. *The Annals of thoracic surgery* 2020, **110**(1):e35-e37.

19. Fredskild MU, Mintz J, Frye MA, McElroy SL, Nolen WA, Kupka R, Grunze H, Keck PE, Jr., Post RM, Kessing LV *et al*: **Adding Increased Energy or Activity to Criterion (A) of the DSM-5 Definition of Hypomania and Mania: Effect on the Diagnoses of 907 Patients From the Bipolar Collaborative Network**. *The Journal of clinical psychiatry* 2019, **80**(6).

20. Gandrup J, Li J, Izadi Z, Gianfrancesco M, Ellingsen T, Yazdany J, Schmajuk G: **Three Quality Improvement Initiatives Improved Performance of Rheumatoid Arthritis Disease Activity Measures in Electronic Health Records: Results from an Interrupted Time Series Study**. *Arthritis care & research* 2019.

21. Gandrup J, Yazdany J: **Using Health Information Technology to Support Use of Patient-Reported Outcomes in Rheumatology**. *Rheumatic diseases clinics of North America* 2019, **45**(2):257-273.

22. Gandrup J, Ali SM, McBeth J, van der Veer SN, Dixon WG: **Remote symptom monitoring integrated into electronic health records: A systematic review**. *Journal of the American Medical Informatics Association : JAMIA* 2020, **27**(11):1752-1763.

23. Gjesing A, Gislason GH, Christensen SB, Jorgensen ME, Merie C, Norgaard ML, Poulsen HE, Gustafsson F, Kober L, Torp-Pedersen C *et al*: **Use of quinine and mortality-risk in patients with heart failure--a Danish nationwide observational study**. *Pharmacoepidemiology and drug safety* 2015, **24**(3):310-318.

24. Grove-Laugesen D, Malmstroem S, Ebbehoj E, Riis AL, Watt T, Hansen KW, Rejnmark L: **Effect of 9 months of vitamin D supplementation on arterial stiffness and blood pressure in Graves' disease: a randomized clinical trial**. *Endocrine* 2019.

25. Grove-Laugesen D, Cramon PK, Malmstroem S, Ebbehoj E, Watt T, Hansen KW, Rejnmark L: **Effects of Supplemental Vitamin D on Muscle Performance and Quality of Life in Graves' Disease: A Randomized Clinical Trial**. *Thyroid : official journal of the American Thyroid Association* 2020, **30**(5):661-671.

26. Grove-Laugesen D, Malmstroem S, Ebbehoj E, Riis AL, Watt T, Rejnmark L, Würgler Hansen K: **Arterial Stiffness and Blood Pressure in Patients Newly Diagnosed with Graves' Disease Compared with Euthyroid Controls**. *European thyroid journal* 2020, **9**(3):148-156.

27. Hansen PW, Gislason GH, Jorgensen ME, Kober L, Jensen PF, Torp-Pedersen C, Andersson C: **Influence of age on perioperative major adverse cardiovascular events and mortality risks in elective non-cardiac surgery**. *European journal of internal medicine* 2016, **35**:55-59.

28. Heftdal LD, Andersen T, Jaehger D, Woetmann A, Ostgard R, Kenngott EE, Syrbe U, Sieper J, Hvid M, Deleuran B *et al*: **Synovial cell production of IL-26 induces bone mineralization in spondyloarthritis**. *Journal of molecular medicine (Berlin, Germany)* 2017, **95**(7):779-787.

29. Heftdal LD, Loft AG, Hendricks O, Ashouri Christiansen A, Schiottz-Christensen B, Arnbak B, Jurik AG, Ostgard R, Winding Deleuran B, Moller HJ *et al*: **Divergent effects on macrophage biomarkers soluble CD163 and CD206 in axial spondyloarthritis**. *Scandinavian journal of clinical and laboratory investigation* 2018, **78**(6):483-489.

30. Heftdal LD, Stengaard-Pedersen K, Ornbjerg LM, Hetland ML, Horslev-Petersen K, Junker P, Ostergaard M, Hvid M, Deleuran B, Moller HJ *et al*: **Soluble CD206 plasma levels in rheumatoid arthritis reflect decrease in disease activity**. *Scandinavian journal of clinical and laboratory investigation* 2017, **77**(5):385-389.

31. Hertle E, Arts IC, van der Kallen CJ, Feskens EJ, Schalkwijk CG, Hoffmann-Petersen IT, Thiel S, Stehouwer CD, van Greevenbroek MM: **Distinct Longitudinal Associations of MBL, MASP-1, MASP-2, MASP-3, and MAp44 With Endothelial Dysfunction and Intima-Media Thickness: The Cohort on Diabetes and Atherosclerosis Maastricht (CODAM) Study**. *Arteriosclerosis, thrombosis, and vascular biology* 2016, **36**(6):1278-1285.

32. Izadi Z, Gandrup J, Katz PP, Yazdany J: **Patient-reported outcome measures for use in clinical trials of SLE: a review**. *Lupus science & medicine* 2018, **5**(1):e000279.

33. Izadi Z, Katz PP, Schmajuk G, Gandrup J, Li J, Gianfrancesco M, Yazdany J: **Effects of Language, Insurance, and Race/Ethnicity on Measurement Properties of the PROMIS Physical Function Short Form 10a in Rheumatoid Arthritis**. *Arthritis care & research* 2019, **71**(7):925-935.

34. Jans O, Bandholm T, Kurbegovic S, Solgaard S, Kjaersgaard-Andersen P, Johansson PI, Kehlet H: **Postoperative anemia and early functional outcomes after fast-track hip arthroplasty: a prospective cohort study**. *Transfusion* 2016, **56**(4):917-925.

35. Jorgensen ME, Andersson C, Norgaard BL, Abdulla J, Shreibati JB, Torp-Pedersen C, Gislason GH, Shaw RE, Hlatky MA: **Functional Testing or Coronary Computed Tomography Angiography in Patients With Stable Coronary Artery Disease**. *Journal of the American College of Cardiology* 2017, **69**(14):1761-1770.

36. Jorgensen ME, Andersson C, Olsen AM, Juel K, Mortensen PE, Jorgensen E, Tilsted HH, von Kappelgaard LM, Torp-Pedersen C, Gislason GH: **Danish trends in pharmacotherapy, comorbidities, and demographics in patients referred for coronary angiography: what changed during a decade?** *European heart journal Cardiovascular pharmacotherapy* 2015, **1**(3):157-165.

37. Jorgensen ME, Andersson C, Vasan RS, Kober L, Abdulla J: **Characteristics and prognosis of heart failure with improved compared with persistently reduced ejection fraction: A systematic review and meta-analyses**. *European journal of preventive cardiology* 2018, **25**(4):366-376.

38. Jorgensen ME, Andersson C, Venkatesan S, Sanders RD: **Beta-blockers in noncardiac surgery: Did observational studies put us back on safe ground?** *British journal of anaesthesia* 2018, **121**(1):16-25.

39. Jorgensen ME, Gislason GH, Andersson C: **Time since stroke and risk of adverse outcomes after surgery--reply**. *Jama* 2014, **312**(18):1930-1931.

40. Jorgensen ME, Hlatky MA, Andersson C: **Association of beta-Blocker Treatment With Adverse Outcomes in Hypertensive Patients Undergoing Noncardiac Surgery--Reply**. *JAMA internal medicine* 2016, **176**(4):563-564.

41. Jorgensen ME, Hlatky MA, Kober L, Sanders RD, Torp-Pedersen C, Gislason GH, Jensen PF, Andersson C: **beta-Blocker-Associated Risks in Patients With Uncomplicated Hypertension Undergoing Noncardiac Surgery**. *JAMA internal medicine* 2015, **175**(12):1923-1931.

42. Jorgensen ME, Sanders RD, Kober L, Mehta K, Torp-Pedersen C, Hlatky MA, Pallisgaard JL, Shaw RE, Gislason GH, Jensen PF *et al*: **Beta-blocker subtype and risks of perioperative adverse events following non-cardiac surgery: a nationwide cohort study**. *European heart journal* 2017, **38**(31):2421-2428.

43. Jorgensen ME, Torp-Pedersen C, Finer N, Caterson I, James WP, Legler UF, Andersson C: **Association between serum bilirubin and cardiovascular disease in an overweight high risk population from the SCOUT trial**. *Nutrition, metabolism, and cardiovascular diseases : NMCD* 2014, **24**(6):656-662.

44. Jorgensen ME, Torp-Pedersen C, Gislason GH, Jensen PF, Berger SM, Christiansen CB, Overgaard C, Schmiegelow MD, Andersson C: **Time elapsed after ischemic stroke and risk of adverse cardiovascular events and mortality following elective noncardiac surgery**. *Jama* 2014, **312**(3):269-277.

45. Kjaer TW, Faurholt-Jepsen D, Medrano R, Elwan D, Mehta K, Christensen VB, Wojcicki JM: **Higher Birthweight and Maternal Pre-pregnancy BMI Persist with Obesity Association at Age 9 in High Risk Latino Children**. *Journal of immigrant and minority health* 2019, **21**(1):89-97.

46. Kjaer TW, Faurholt-Jepsen D, Mehta KM, Christensen VB, Epel E, Lin J, Blackburn E, Wojcicki JM: **Shorter preschool, leukocyte telomere length is associated with obesity at age 9 in Latino children**. *Clinical obesity* 2018, **8**(2):88-94.

47. Kjaer TW, Medrano R, Wojcicki JM: **Rapid infant weight gain and prenatal maternal depression are associated with prehypertension/hypertension before age 9**. *Journal of human hypertension* 2020, **34**(11):795-801.

48. Kragstrup TW, Adams M, Lomholt S, Nielsen MA, Heftdal LD, Schafer P, Deleuran B: **IL-12/IL-23p40 identified as a downstream target of apremilast in ex vivo models of arthritis**. *Therapeutic advances in musculoskeletal disease* 2019, **11**:1759720x19828669.

49. Kragstrup TW, Andersen T, Heftdal LD, Hvid M, Gerwien J, Sivakumar P, Taylor PC, Senolt L, Deleuran B: **The IL-20 Cytokine Family in Rheumatoid Arthritis and Spondyloarthritis**. *Frontiers in immunology* 2018, **9**:2226.

50. Kurbegovic S, Andersen J, Krenk L, Kehlet H: **Delirium in fast-track colonic surgery**. *Langenbeck's archives of surgery* 2015, **400**(4):513-516.

51. Kurbegovic S, Berg KD, Thomsen FB, Gruschy L, Iversen P, Brasso K, Roder MA: **The risk of biochemical recurrence for intermediate-risk prostate cancer after radical prostatectomy**. *Scandinavian journal of urology* 2017, **51**(6):450-456.

52. Kurbegovic S, Juhl K, Chen H, Qu C, Ding B, Leth JM, Drzewiecki KT, Kjaer A, Cheng Z: **Molecular Targeted NIR-II Probe for Image-Guided Brain Tumor Surgery**. *Bioconjugate chemistry* 2018, **29**(11):3833-3840.

53. Lassen MCH, Biering-Sorensen SR, Olsen FJ, Skaarup KG, Tolstrup K, Qasim AN, Mogelvang R, Jensen JS, Biering-Sorensen T: **Ratio of transmitral early filling velocity to early diastolic strain rate predicts long-term risk of cardiovascular morbidity and mortality in the general population**. *European heart journal* 2019, **40**(6):518-525.

54. Lassen MCH, Jensen MT, Biering-Sorensen T, Mogelvang R, Fritz-Hansen T, Vilsboll T, Rossing P, Jorgensen PG: **Prognostic value of ratio of transmitral early filling velocity to early diastolic strain rate in patients with Type 2 diabetes**. *European heart journal cardiovascular Imaging* 2019, **20**(10):1171-1178.

55. Lassen MCH, Qasim AN, Biering-Sorensen T, Reeh JLT, Watnick T, Seliger SL, Chen H, Sawan MA, Nguyen D, Li Y *et al*: **Cardiac function assessed by myocardial deformation in adult polycystic kidney disease patients**. *BMC nephrology* 2019, **20**(1):324.

56. Lassen MCH, Sengelov M, Qasim A, Jorgensen PG, Bruun NE, Olsen FJ, Fritz-Hansen T, Gislason G, Biering-Sorensen T: **Ratio of Transmitral Early Filling Velocity to Early Diastolic Strain Rate Predicts All-Cause Mortality in Heart Failure with Reduced Ejection Fraction**. *Journal of cardiac failure* 2019.

57. Lassen MCH, Skaarup KG, Iversen AZ, Jorgensen PG, Olsen FJ, Galatius S, Biering-Sorensen T: **Ratio of Transmitral Early Filling Velocity to Early Diastolic Strain Rate as a Predictor of Cardiovascular Morbidity and Mortality Following Acute Coronary Syndrome**. *The American journal of cardiology* 2019, **123**(11):1776-1782.

58. Lassen MCH, Biering-Sørensen T, Jørgensen PG, Andersen HU, Rossing P, Jensen MT: **Sex differences in the association between myocardial function and prognosis in type 1 diabetes without known heart disease: the Thousand & 1 Study**. *European heart journal cardiovascular Imaging* 2020.

59. Lassen MCH, Olsen FJ, Skaarup KG, Tolstrup K, Qasim AN, Gislason G, Biering-Sørensen T: **The clinical application of the ratio of transmitral early filling velocity to early diastolic strain rate: a systematic review and meta-analysis**. *Journal of echocardiography* 2020, **18**(2):94-104.

60. Lassen MCH, Skaarup KG, Lind JN, Alhakak AS, Sengeløv M, Nielsen AB, Espersen C, Ravnkilde K, Hauser R, Schöps LB *et al*: **Echocardiographic abnormalities and predictors of mortality in hospitalized COVID-19 patients: the ECHOVID-19 study**. *ESC heart failure* 2020.

61. Lassen MCH, Skaarup KG, Sengeløv M, Iversen K, Ulrik CS, Jensen JUS, Biering-Sørensen T: **Alcohol Consumption and the Risk of Acute Respiratory Distress Syndrome in COVID-19**. *Annals of the American Thoracic Society* 2020.

62. Loft MD, Berg KD, Kjaer A, Iversen P, Ferrari M, Zhang CA, Brasso K, Brooks JD, Roder MA: **Temporal Trends in Clinical and Pathological Characteristics for Men Undergoing Radical Prostatectomy Between 1995 and 2013 at Rigshospitalet, Copenhagen, Denmark, and Stanford University Hospital, United States**. *Clinical genitourinary cancer* 2017.

63. Loft MD, Sun Y, Liu C, Christensen C, Huang D, Kjaer A, Cheng Z: **Improved positron emission tomography imaging of glioblastoma cancer using novel (68)Ga-labeled peptides targeting the urokinase-type plasminogen activator receptor (uPAR)**. *Amino acids* 2017, **49**(6):1089-1100.

64. Malmborg M, Schmiegelow MDS, Nørgaard CH, Munch A, Gerds T, Schou M, Kistorp C, Torp-Pedersen C, Hlatky MA, Gislason G: **Does type 2 diabetes confer higher relative rates of cardiovascular events in women compared with men?** *European heart journal* 2020, **41**(13):1346-1353.

65. Malmstroem S, Grove-Laugesen D, Riis AL, Bruun BJ, Ebbehoj E, Hansen KW, Watt T, Rejnmark L: **Muscle Performance and Postural Stability Are Reduced in Patients with Newly Diagnosed Graves' Disease**. *Thyroid : official journal of the American Thyroid Association* 2019, **29**(6):783-789.

66. Malmstroem S, Rejnmark L, Imboden JB, Shoback DM, Bikle DD: **Current Assays to Determine Free 25-Hydroxyvitamin D in Serum**. *Journal of AOAC International* 2017, **100**(5):1323-1327.

67. McLaughlin JE, Wolcott MD, Hubbard D, Umstead K, Rider TR: **A qualitative review of the design thinking framework in health professions education**. *BMC medical education* 2019, **19**(1):98.

68. Modin D, Claggett B, Sindet-Pedersen C, Lassen MCH, Skaarup KG, Jensen JUS, Fralick M, Schou M, Lamberts M, Gerds T *et al*: **Acute COVID-19 and the Incidence of Ischemic Stroke and Acute Myocardial Infarction**. *Circulation* 2020, **142**(21):2080-2082.

69. Morita S, Villalta SA, Feldman HC, Register AC, Rosenthal W, Hoffmann-Petersen IT, Mehdizadeh M, Ghosh R, Wang L, Colon-Negron K *et al*: **Targeting ABL-IRE1alpha Signaling Spares ER-Stressed Pancreatic beta Cells to Reverse Autoimmune Diabetes**. *Cell metabolism* 2017, **25**(5):1207.

70. Morita S, Villalta SA, Feldman HC, Register AC, Rosenthal W, Hoffmann-Petersen IT, Mehdizadeh M, Ghosh R, Wang L, Colon-Negron K *et al*: **Targeting ABL-IRE1alpha Signaling Spares ER-Stressed Pancreatic beta Cells to Reverse Autoimmune Diabetes**. *Cell metabolism* 2017, **25**(4):883-897.e888.

71. Nielsen AB, Zhou M, de Smith AJ, Wang R, McCoy L, Hansen H, Morimoto L, Gronbaek K, Johansen C, Kogan SC *et al*: **Increased neonatal level of arginase 2 in cases of childhood acute lymphoblastic leukemia implicates immunosuppression in etiology**. *Haematologica* 2019.

72. Nielsen AB, Skaarup KG, Lassen MCH, Djernæs K, Hansen ML, Svendsen JH, Johannessen A, Hansen J, Sørensen SK, Gislason G *et al*: **Usefulness of left atrial speckle tracking echocardiography in predicting recurrence of atrial fibrillation after radiofrequency ablation: a systematic review and meta-analysis**. *The international journal of cardiovascular imaging* 2020, **36**(7):1293-1309.

73. Norgaard CH, Jakobsen LH, Gentles AJ, Dybkaer K, El-Galaly TC, Bodker JS, Schmitz A, Johansen P, Herold T, Spiekermann K *et al*: **Subtype assignment of CLL based on B-cell subset associated gene signatures from normal bone marrow - A proof of concept study**. *PloS one* 2018, **13**(3):e0193249.

74. Norgaard CH, Mosslemi M, Lee CJ, Torp-Pedersen C, Wong ND: **The Importance and Role of Multiple Risk Factor Control in Type 2 Diabetes**. *Current cardiology reports* 2019, **21**(5):35.

75. Norgaard CH, Sogaard NB, Biccler JL, Pilgaard L, Eskesen MH, Kjartansdottir TH, Bogsted M, El-Galaly TC: **Limited value of routine follow-up visits in chronic lymphocytic leukemia managed initially by watch and wait: A North Denmark population-based study**. *PloS one* 2018, **13**(12):e0208180.

76. Ostergaard JA, Ruseva MM, Malik TH, Hoffmann-Petersen IT, Pickering MC, Thiel S, Hansen TK: **Increased Autoreactivity of the Complement-Activating Molecule Mannan-Binding Lectin in a Type 1 Diabetes Model**. *Journal of diabetes research* 2016, **2016**:1825738.

77. Ostergaard JA, Thiel S, Hoffmann-Petersen IT, Hovind P, Parving HH, Tarnow L, Rossing P, Hansen TK: **Incident microalbuminuria and complement factor mannan-binding lectin-associated protein 19 in people with newly diagnosed type 1 diabetes**. *Diabetes/metabolism research and reviews* 2017, **33**(5).

78. Ovesen S, Durack J, Kirk KF, Nielsen HL, Nielsen H, Lynch SV: **Motility and biofilm formation of the emerging gastrointestinal pathogen Campylobacter concisus differs under microaerophilic and anaerobic environments**. *Gut microbes* 2019, **10**(1):34-44.

79. Ovesen S, Kirk KF, Nielsen HL, Nielsen H: **Motility of Campylobacter concisus isolated from saliva, feces, and gut mucosal biopsies**. *APMIS : acta pathologica, microbiologica, et immunologica Scandinavica* 2017, **125**(3):230-235.

80. Ragouzeos D, Gandrup J, Berrean B, Li J, Murphy M, Trupin L, Yazdany J, Schmajuk G: **"Am I OK?" using human centered design to empower rheumatoid arthritis patients through patient reported outcomes**. *Patient education and counseling* 2019, **102**(3):503-510.

81. Rasmussen BS, Sorensen CL, Kurbegovic S, Orholt M, Talman MM, Herly M, Pipper CB, Kolle ST, Rangatchew F, Holmgaard R *et al*: **Cell-Enriched Fat Grafting Improves Graft Retention in a Porcine Model: A Dose-Response Study of Adipose-Derived Stem Cells versus Stromal Vascular Fraction**. *Plastic and reconstructive surgery* 2019, **144**(3):397e-408e.

82. Rasmussen BS, Sorensen CL, Vester-Glowinski PV, Herly M, Kurbegovic S, Orholt M, Svalgaard JD, Kolle ST, Kristensen AT, Talman MM *et al*: **A Novel Porcine Model for Future Studies of Cell-enriched Fat Grafting**. *Plastic and reconstructive surgery Global open* 2018, **6**(4):e1735.

83. Roder MA, Berg KD, Loft MD, Thomsen FB, Ferrari M, Kurbegovic S, Rytgaard HC, Gruschy L, Brasso K, Gerds TA *et al*: **The CPC Risk Calculator: A New App to Predict Prostate-specific Antigen Recurrence During Follow-up After Radical Prostatectomy**. *European urology focus* 2018, **4**(3):360-368.

84. Sondergaard MM, Nielsen JB, Mortensen RN, Gislason G, Kober L, Lippert F, Graff C, Haunso S, Svendsen JH, Kragholm KH *et al*: **Associations between common ECG abnormalities and out-of-hospital cardiac arrest**. *Open heart* 2019, **6**(1):e000905.

85. Søndergaard MM, Hlatky MA, Stefanick ML, Vittinghoff E, Nah G, Allison M, Gemmill A, Van Horn L, Park K, Salmoirago-Blotcher E *et al*: **Association of Adverse Pregnancy Outcomes With Risk of Atherosclerotic Cardiovascular Disease in Postmenopausal Women**. *JAMA cardiology* 2020, **5**(12):1390-1398.

86. Stevens EM, Vladar EK, Alanin MC, Christensen ST, von Buchwald C, Milla C: **Ciliary Localization of the Intraflagellar Transport Protein IFT88 Is Disrupted in Cystic Fibrosis**. *American journal of respiratory cell and molecular biology* 2020, **62**(1):120-123.

87. Swaminathan S, Hansen AS, Heftdal LD, Dhanasekaran R, Deutzmann A, Fernandez WDM, Liefwalker DF, Horton C, Mosley A, Liebersbach M *et al*: **MYC functions as a switch for natural killer cell-mediated immune surveillance of lymphoid malignancies**. *Nature communications* 2020, **11**(1):2860.

88. Underbjerg L, Malmstroem S, Sikjaer T, Rejnmark L: **Bone Status Among Patients With Nonsurgical Hypoparathyroidism, Autosomal Dominant Hypocalcaemia, and Pseudohypoparathyroidism: A Cohort Study**. *Journal of bone and mineral research : the official journal of the American Society for Bone and Mineral Research* 2018, **33**(3):467-477.

89. Venkatesan S, Jorgensen ME, Manning HJ, Andersson C, Mozid AM, Coburn M, Moonesinghe SR, Foex P, Mythen M, Grocott MPW *et al*: **Preoperative chronic beta-blocker prescription in elderly patients as a risk factor for postoperative mortality stratified by preoperative blood pressure: a cohort study**. *British journal of anaesthesia* 2019, **123**(2):118-125.

90. Venkatesan S, Myles PR, Manning HJ, Mozid AM, Andersson C, Jorgensen ME, Hardman JG, Moonesinghe SR, Foex P, Mythen M *et al*: **Cohort study of preoperative blood pressure and risk of 30-day mortality after elective non-cardiac surgery**. *British journal of anaesthesia* 2017, **119**(1):174.

91. Waaijer CJF, Ommering BWC, van der Wurff LJ, van Leeuwen TN, Dekker FW: **Scientific activity by medical students: the relationship between academic publishing during medical school and publication careers after graduation**. *Perspectives on medical education* 2019, **8**(4):223-229.

92. Zareini B, Blanche P, D'Souza M, Elmegaard Malik M, Nørgaard CH, Selmer C, Gislason G, Kristensen SL, Køber L, Torp-Pedersen C *et al*: **Type 2 Diabetes Mellitus and Impact of Heart Failure on Prognosis Compared to Other Cardiovascular Diseases: A Nationwide Study**. *Circulation Cardiovascular quality and outcomes* 2020, **13**(7):e006260.

93. Olsen FJ, Lassen MCH, Brainin P, Bech J, Alhakak AS, Pedersen S, Claggett B, Fritz-Hansen T, Folke F, Gislason GH *et al*: **Myocardial performance index is associated with cardiac computed tomography findings in patients with suspected coronary artery disease**. *Echocardiography (Mount Kisco, NY)* 2020, **37**(11):1741-1748.

94. Skaarup KG, Lassen MCH, Lind JN, Alhakak AS, Sengeløv M, Nielsen AB, Espersen C, Hauser R, Schöps LB, Holt E *et al*: **Myocardial Impairment and Acute Respiratory Distress Syndrome in Hospitalized Patients With COVID-19: The ECHOVID-19 Study**. *JACC Cardiovascular imaging* 2020, **13**(11):2474-2476.

95. Skaarup KG, Lassen MCH, Marott JL, Biering-Sørensen SR, Jørgensen PG, Appleyard M, Berning J, Høst N, Jensen G, Schnohr P *et al*: **The impact of cardiovascular risk factors on global longitudinal strain over a decade in the general population: the copenhagen city heart study**. *The international journal of cardiovascular imaging* 2020, **36**(10):1907-1916.

Appendix 1. References for publications of DARE Fellows and Alumni, 2015-2020, Cohorts 1-5

All (97)

[1-92]

First author (17/24 students; 52/97-54% publications)

[7, 14-22, 28-30, 35-43, 45, 46, 50-63, 65, 66, 71-73, 78, 84-86]

Collaborative author (43/97- 46% publications)

[1-6, 8-13, 23-27, 31-34, 48, 49, 64, 67, 69, 70, 76, 77, 80-83, 87-95]

Joint US-DANISH publications (14/24 students; 14/97-14% publications)

[35, 40-42, 52, 55, 56, 62, 63, 66, 71, 73, 78, 83]
